# Supplementary figures and images for: Quantitative Trait Locus Analysis of Hessian Fly Resistance in Soft Red Winter Wheat
Source: Genes (Basel). 2023 Sep 17;14(9):1812. doi: 10.3390/genes14091812 (PMC10531203; doi:10.3390/genes14091812)

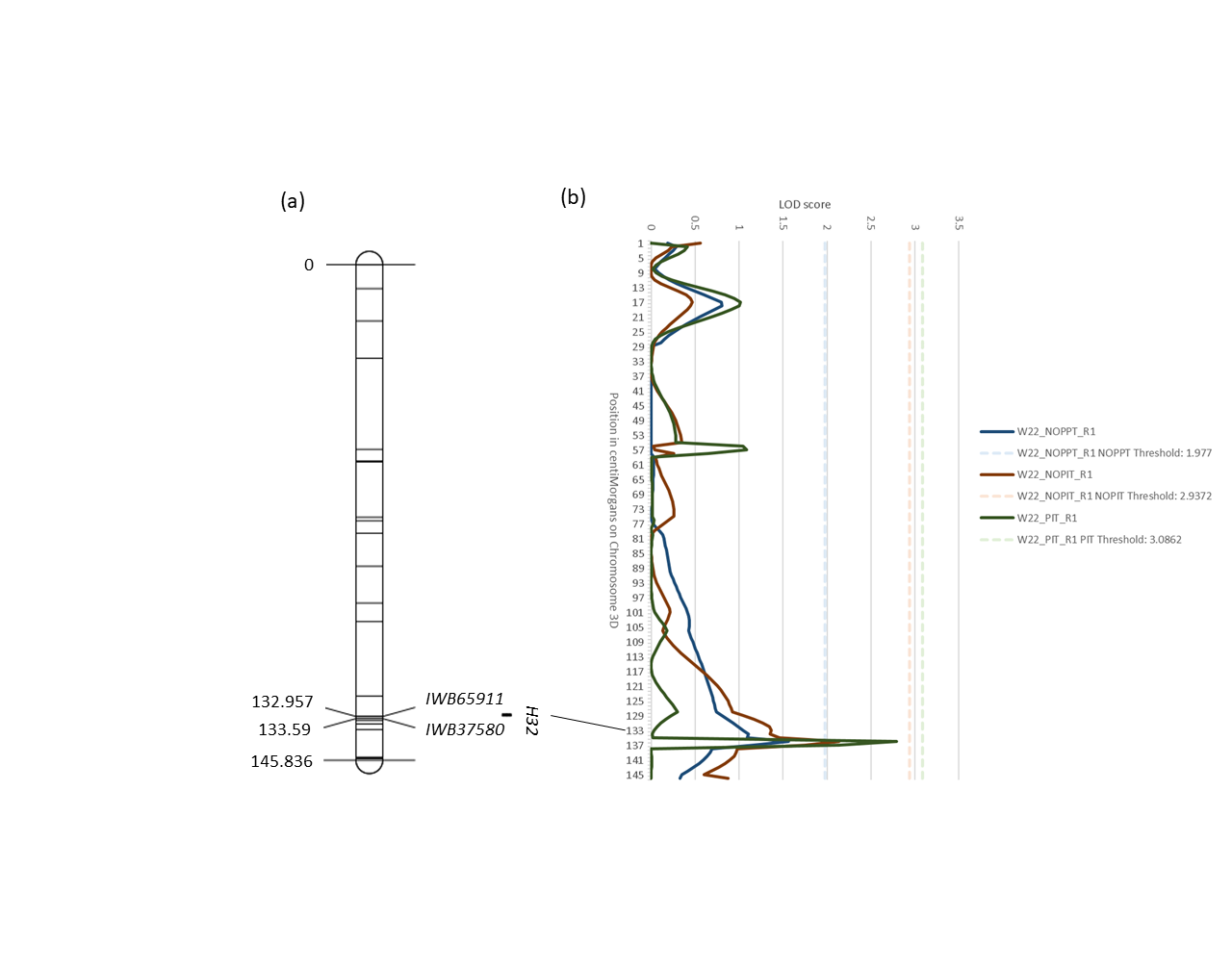

Supplement: Supplementary file 1 [file genes-14-01812-s001.zip › H32 Figure S1.tif]

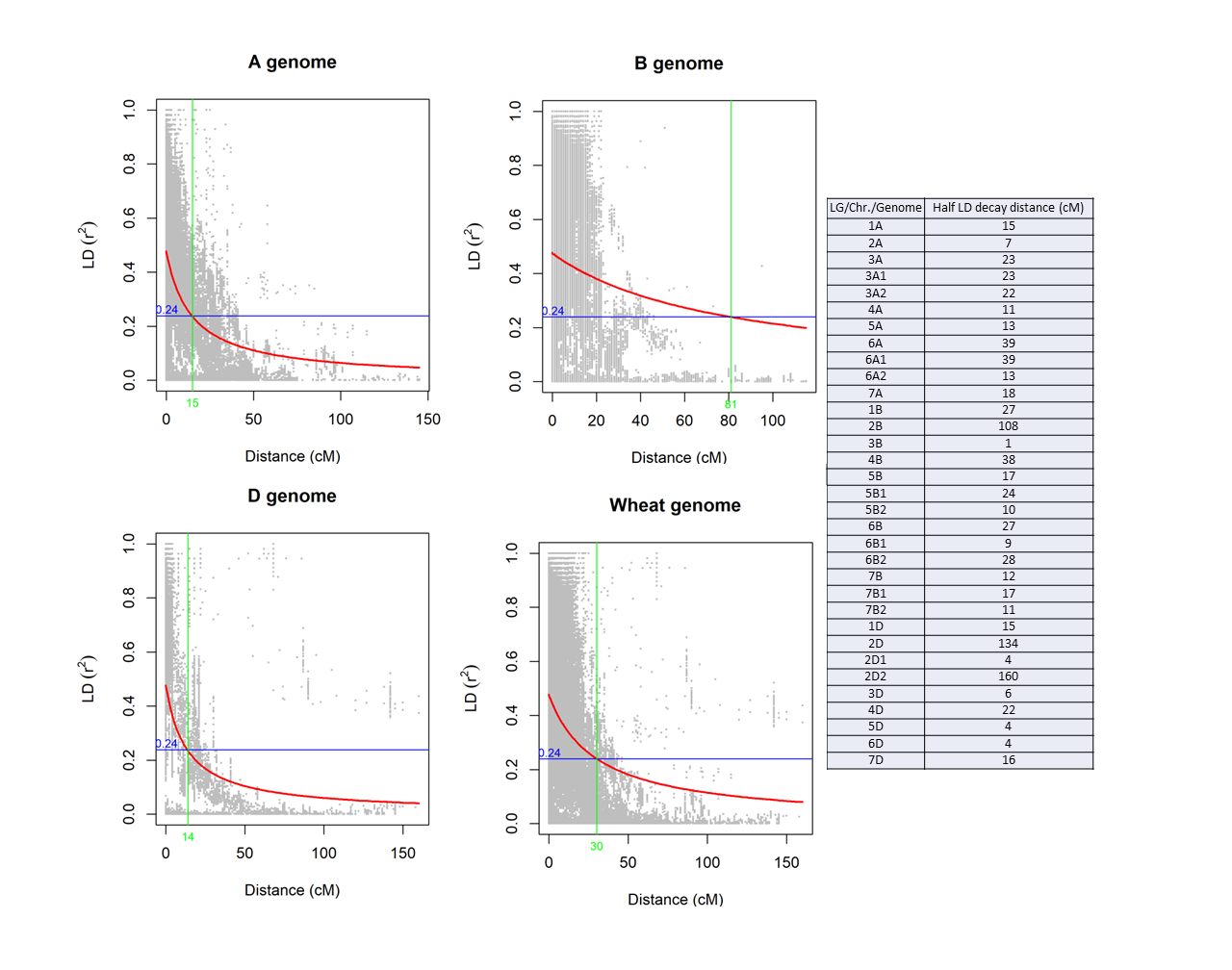

Supplement: Supplementary file 1 [file genes-14-01812-s001.zip › H32 Figure S2.tif]
